# Complement receptors C5aR1 and C5aR2 act differentially during the early immune response after bone fracture but are similarly involved in bone repair

**DOI:** 10.1038/s41598-017-14444-3

**Published:** 2017-10-25

**Authors:** Anna Kovtun, Stephanie Bergdolt, Yvonne Hägele, Rebekka Matthes, John D. Lambris, Markus Huber-Lang, Anita Ignatius

**Affiliations:** 1 Institute of Orthopaedic Research and Biomechanics, Centre for Trauma Research Ulm, University of Ulm, D-89081 Ulm, Germany; 2 Department of Pathology and Laboratory Medicine, University of Pennsylvania School of Medicine, Philadelphia, PA 19104-6100 USA; 3 Institute of Clinical and Experimental Trauma Immunology (ITI), Ulm University Hospital, D-89081 Ulm, Germany

## Abstract

Severely injured patients frequently suffer compromised fracture healing because of systemic post-traumatic inflammation. An important trigger of the posttraumatic immune response is the complement anaphylatoxin C5a, which acts via two receptors, C5aR1 and C5aR2, expressed on immune and bone cells. The blockade of C5a-mediated inflammation during the early inflammatory phase was demonstrated to improve fracture healing after severe injury. However, the distinct roles of the two complement receptors C5aR1 and C5aR2 in bone has to date not been studied. Here, we investigated bone turnover and regeneration in mice lacking either C5aR1 or C5aR2 in a model of isolated fracture and after severe injury, combining the fracture with an additional thoracic trauma. Both C5aR1^−/−^ and C5aR2^−/−^ mice displayed an increased bone mass compared to wild-type controls due to reduced osteoclast formation and increased osteoblast numbers, respectively. Following fracture, the inflammatory response was differently affected in these strains: It was decreased in C5aR1^−/−^ mice but enhanced in C5aR2^−/−^ mice. Both strains exhibited impaired fracture healing, disturbed osteoclastogenesis and delayed cartilage-to-bone transformation. Thus, our data suggest that C5aR1 and C5aR2 differentially regulate the immune response after fracture and are required for effective cartilage-to-bone transformation in the fracture callus and for undisturbed bone healing.

## Introduction

The complement system is an important part of the innate immune system. It includes more than 40 proteases, which are sequentially activated after inflammatory stimuli. Activation of the complement system leads, amongst others, to the generation of the potent anaphylatoxin C5a^1–3^. C5a is a strong chemoattractant and an effective inflammatory mediator, which, upon binding to its G protein-coupled membrane-bound receptor C5aR1 (C5R1, C5aR, CD88) provokes and amplifies inflammatory reactions by inducing degranulation, cytokine release and oxidative burst of immune cells^4^. C5a can also bind to its second receptor, C5aR2 (C5a receptor-like 2, C5L2, GPR77), the function of which continues to be strongly debated^5^. C5aR2 is un-coupled from G proteins and is, therefore, generally described as a modulator of C5aR1 signalling, exerting either anti- or pro-inflammatory activities^6–8^. By controlling the availability of C5a, C5aR2 might, on the one hand, limit C5a/C5aR1-mediated cell activation, and thereby act anti-inflammatory. On the other hand, C5aR2 may induce G protein-independent activation of immune cells, thus exerting pro-inflammatory effects^5^.

In recent years, evidence has accumulated that the complement system plays a crucial role in bone metabolism and contributes to inflammatory bone disorders^9,10^. Bone tissue is continuously rebuilt during bone remodelling, a dynamic balance between bone-forming osteoblasts and resorbing osteoclasts, which is essential for maintaining bone mass. Complement might regulate this process, because it was demonstrated that complement protein C3 is produced by osteoblasts and potentiates osteoclast formation *in vitro* ^11,12^. Our group showed that osteoblasts and osteoclasts are not only able to produce C3 and/or C5, but to cleave C5 and produce biologically active C5a^13^. Furthermore, C3aR and C5aR1 are strongly up-regulated during osteoblastogenesis and mediate the production of pro-inflammatory cytokines, including interleukin (IL)-6 and IL-8 in osteoblasts^13–15^. These data indicate that the activation of the C3aR- and C5aR1-axis induces an immune response in these cells. Therefore, anaphylatoxin receptor signalling in bone might be particularly important under inflammatory conditions. Confirming this, C3aR- and C5aR1-deficient mice are protected against inflammatory arthritis^16^, and bone loss in periodontitis is associated with increased C5aR1 activity^17^. Our group demonstrated strong C5aR1 up-regulation by osteoblasts in response to bone injury and that bone healing is severely disturbed in mice with osteoblast-specific C5aR1 overexpression, suggesting that osteoblasts are effector cells for C5a in bone repair^14,18^. Furthermore, the blockade of C5a/C5aR1 signalling using a specific receptor antagonist in the early posttraumatic phase significantly improved compromised fracture healing in a rodent model of severe trauma, indicating that excessive complement activation in posttraumatic inflammation may negatively affect bone regeneration^19^. By contrast, bone repair was impaired in mice lacking C5^20^, suggesting that it may be critical to maintain balanced C5a actions during bone healing. Thereby, the second anaphylatoxin receptor C5aR2 might play a modulatory role. However, to best of our knowledge, this receptor has to date not been investigated in bone.

This study aims to investigate the roles of both C5a receptors in bone fracture healing using C5aR1^−/−^ and C5aR2^−/−^ mice. We used an isolated fracture model and model of severe trauma, where the fracture was combined with an additional thoracic trauma to induce a more pronounced inflammation^21^.

## Results

### Bone phenotype of C5aR1^−/−^ and C5aR2^−/−^ mice

**Figure 1:**
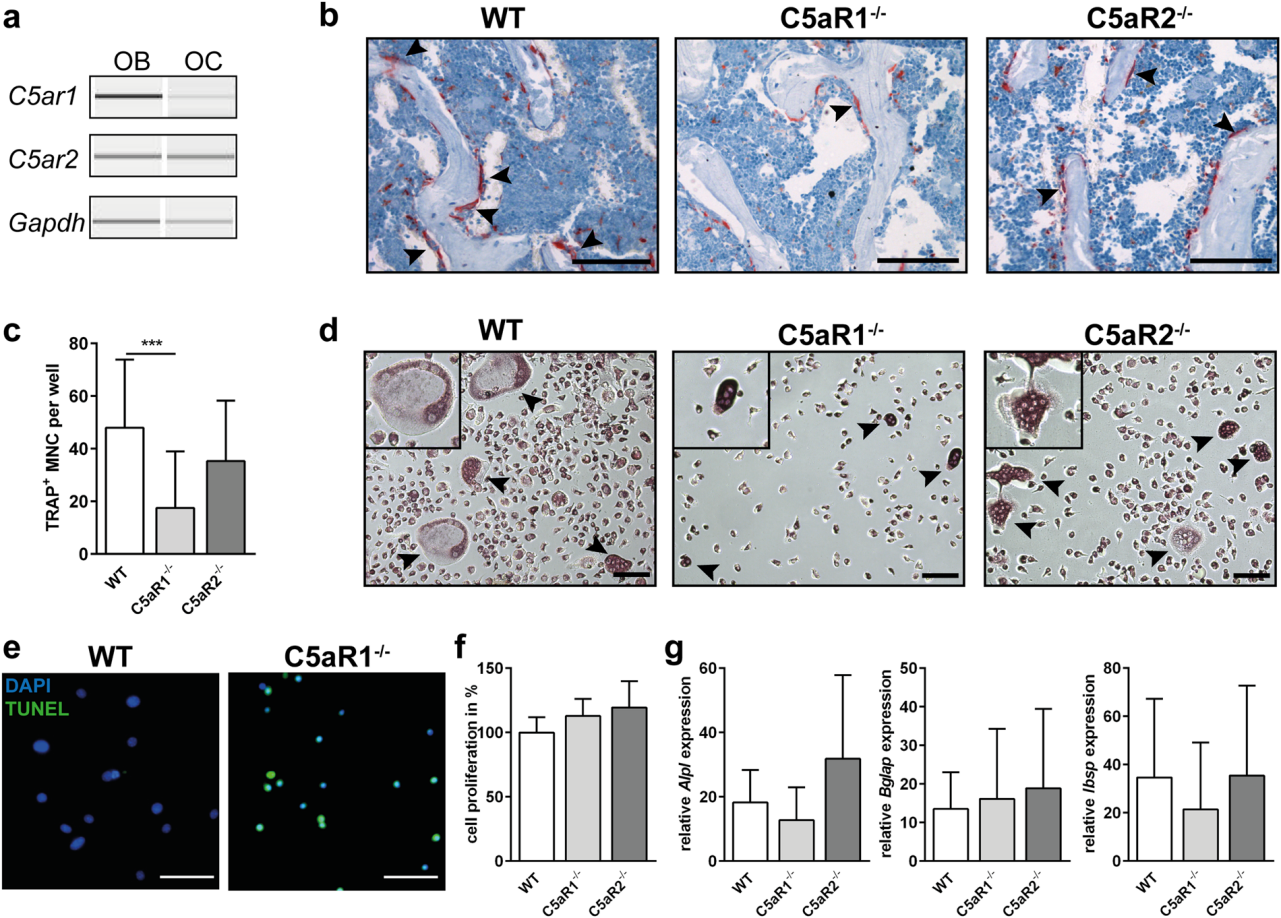
Gene expression of C5aR1 and C5aR2 in primary osteoblasts (OB) and osteoclasts (OC) (**a**). Representative images of tartrate-resistant acid phosphatase (TRAP)-positive osteoclasts in spine (**b**), arrowheads indicate osteoclasts, scale bar 100 µm. *In vitro* osteogenic differentiation and proliferation of primary osteoblasts and osteoclasts isolated from long bones (**c**–**g**). Quantitative analysis of TRAP-positive multi-nucleated cells (MNC) (**c**), n = 12-20, ***p < 0.001. Staining for TRAP in bone marrow-isolated osteoclast progenitor cells after differentiation (**d**), arrowheads indicate osteoclasts, scale bar 100 µm. TUNEL staining in bone marrow-isolated osteoclast progenitor cells after differentiation (**e**), scale bar 50 µm. BrdU proliferation assay of primary osteoblasts (**f**), n = 4–7. Expression of osteogenic marker genes in primary osteoblasts after 14 days of differentiation (**g**), n = 5–7, data were not significantly different, *p < 0.05.

First, we analysed the bone phenotype of C5aR1- and C5aR2-knockout mice and the corresponding wild-type (WT) mice using a non-destructive three-point bending test, micro-computed tomography (µCT) and dynamic histomorphometry (Table 1). Additionally, to check whether the absence of C5aRs could affect bone directly, we analysed the expression of C5aR1 and C5aR2 in bone cells, showing that in WT mice osteoblasts and osteoclasts expressed both receptors (Fig. 1a).

**Table 1:** Bone phenotype of 12-week-old C5aR1- and C5aR2-knockout mice.

	Parameters	WT	C5aR1^−/−^	C5aR2^−/−^
Cortical bone	EI (Nmm^2^)	2647 ± 241	4032 ± 657*	3405 ± 800
	TMD (mg/cm^3^ of HA)	1153 ± 27	1328 ± 16*	1275 ± 23*
	Ix (mm^4^)	0.15 ± 0.03	0.21 ± 0.03*	0.16 ± 0.04
	C.Th (mm)	0.16 ± 0.02	0.20 ± 0.01*	0.17 ± 0.01
Trabecular bone	BV/TV (%)	24.3 ± 3.5	34.3 ± 2.6*	30.6 ± 3.7*
	TMD (mg/cm^3^ of HA)	756 ± 41	806 ± 40	788 ± 31
	Tb.N (1/mm)	4.6 ± 0.2	5.1 ± 0.3*	4.9 ± 0.4
	Tb.Th (mm)	0.05 ± 0.01	0.07 ± 0.01*	0.06 ± 0.00*
	Tb.Sp (mm)	0.17 ± 0.01	0.15 ± 0.01*	0.16 ± 0.01*
	Ob.N/B.Pm (1/mm)	13.6 ± 3.4	14.4 ± 2.5	16.3 ± 2.7*
	Oc.N/B.Pm (1/mm)	2.3 ± 0.4	1.1 ± 0.2*	2.3 ± 0.5
	BFR/BS (µm^3^/µm^2^/t)	0.4 ± 0.1	0.6 ± 0.1*	0.3 ± 0.1
Serum	CTX (ng/mL)	21.3 ± 9.8	25.5 ± 5.5	16.8 ± 2.7
	PINP (ng/mL)	6.5 ± 1.8	10.0 ± 2.9*	6.1 ± 1.4

N = 5, *p < 0.05 compared to WT mice. EI: bending stiffness, TMD: tissue mineral density, Ix: moment of inertia, C.Th: cortical thickness, BV/TV: bone volume per total volume, Tb.N: trabecular number, Tb.Th: trabecular thickness, Tb.Sp: trabecular separation, Ob.N/B.Pm: osteoblast number per bone perimeter, Oc.N/B.Pm: osteoclast number per bone perimeter, BFR/BS: bone formation rate per bone surface, CTX: C-terminal telopeptide, PINP: procollagen type I N-terminal propeptide.

C5aR1^−/−^ mice displayed a significantly increased bone mass. In the cortical compartment of the femur, cortical thickness, moment of inertia and tissue mineralization were increased, resulting in an enhanced bending stiffness. The trabecular bone content, trabecular number and thickness, the bone formation rate, and the systemic concentration of procollagen type I N-terminal propeptide (PINP), a marker for bone formation, were increased. Osteoclast numbers were decreased, but serum levels of C-terminal telopeptide (CTX) were not significantly affected. Thus, the *in vivo* results do not clearly reveal whether the increased bone mass of C5aR1^−/−^ mice results from increased osteoblast or decreased osteoclast activity or both (Table 1 and Fig. 1b). However, *in vitro* analyses of primary cells isolated from C5aR1^−/−^ mice demonstrated that osteoclast formation was considerably disturbed, confirming low osteoclast numbers *in vivo* (Fig. 1c,d). The low osteoclast numbers might result from increased apoptosis of osteoclast-progenitor cells, as demonstrated by TUNEL assay (Fig. 1e). In contrast, osteoblast proliferation and the expression of osteogenic marker genes *Alpl*, *Ibsp* or *Bglap* were not significantly affected (Fig. 1f,g). These results suggest that the increased bone mass of C5aR1^−/−^ mice may rather result from disturbed bone resorption. Tissue mineral density (TMD) in the trabecular compartment was not significantly changed, indicating unaffected mineralization of bone matrix (Table 1).

C5aR2^−/−^ mice also displayed an increased bone mass, albeit the phenotype was less pronounced, as shown by unaffected systemic levels of bone turnover markers CTX and PINP. Osteoclast numbers were not decreased; instead, osteoblast numbers were increased. *Ex vivo*, osteoclast formation as well as osteoblast proliferation and differentiation were not significantly affected (Fig. 1c,f,g). These results indicate that the increased bone mass might result from slightly increased osteoblast numbers *in vivo*.

### Inflammatory response in C5aR1^−/−^ and C5aR2^−/−^ mice after injury

#### Serum

**Figure 2:**
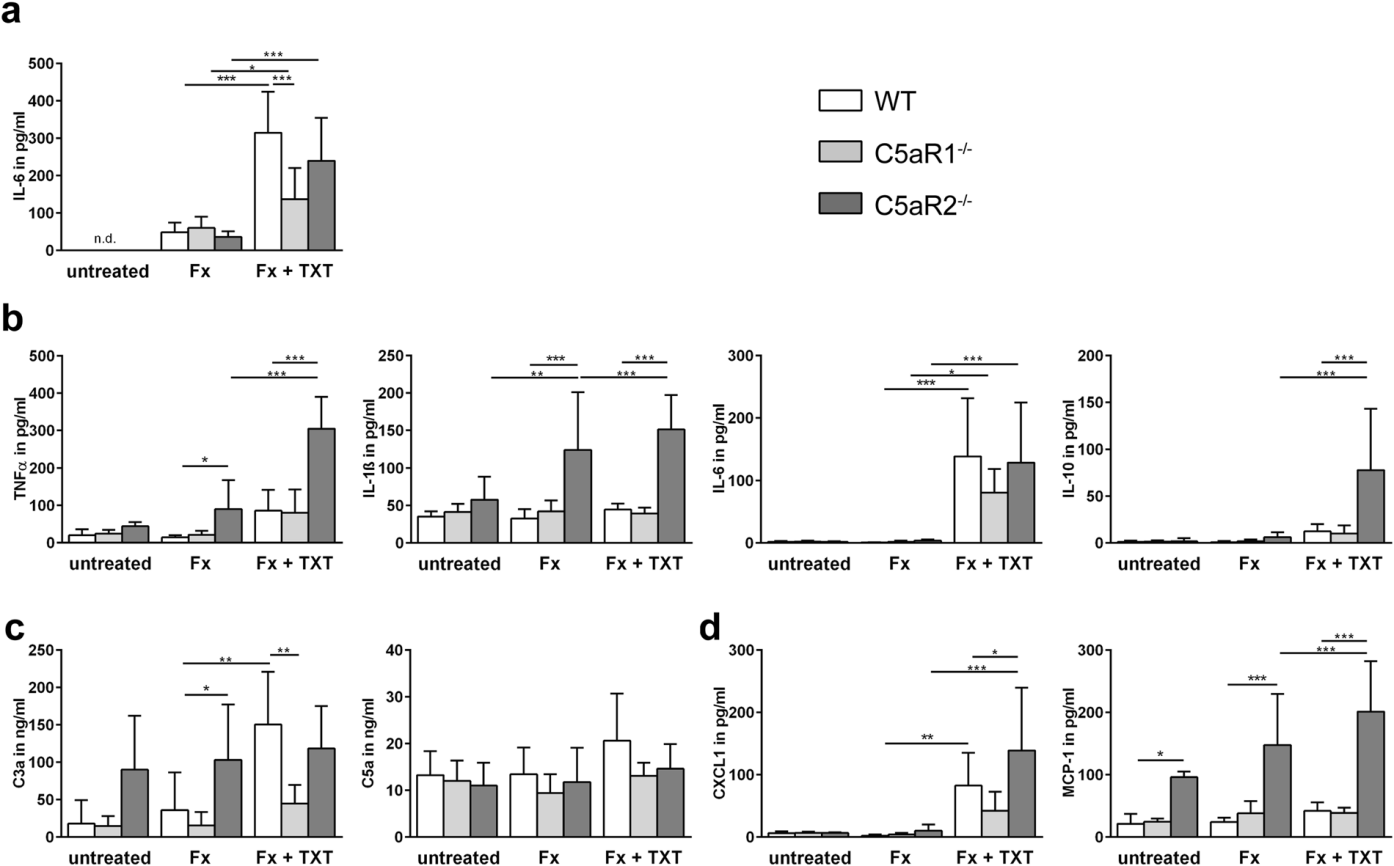
Inflammatory cytokine and chemokine concentrations in serum and bronchoalveolar lavage (BAL) fluid 3 h after trauma. Serum IL-6 concentrations (**a**), n = 4–6. Concentration of inflammatory cytokines (**b**), anaphylatoxins (**c**) and chemokines (**d**) in BAL fluids 3 h after trauma, n = 4–5 for untreated mice, n = 5–8 for mice with fracture or combined trauma. Fx: mice with isolated fracture, Fx + TXT: mice with combined fracture and thoracic trauma. *p < 0.05, **p < 0.01, ***p < 0.001.

In all untreated mouse strains, IL-6 serum concentrations were below the detection limit (Fig. 2a). The isolated fracture did not affect serum IL-6 levels, whereas the combined fracture and thoracic trauma significantly increased IL-6 concentrations in all mouse strains, indicating systemic inflammation. However, C5aR1^−/−^ mice displayed a significantly reduced increase of systemic IL-6 compared to the WT mice.

#### Bronchoalveolar lavage (BAL) fluid

As expected, after isolated fracture, the concentrations of inflammatory mediators in BAL fluid of WT and C5aR1^−/−^ mice did not significantly differ from untreated controls (Fig. 2b–d). However, in C5aR2^−/−^ mice, the concentrations of IL-1β, tumour necrosis factor (TNF) α and monocyte chemoattractant protein-1 (MCP-1) were significantly elevated after isolated fracture, indicating a pulmonary inflammatory response after bone fracture even in the absence of thoracic trauma. Moreover, MCP-1 was increased in BAL fluid of C5aR2^−/−^ mice without any injury, presumably because of general anaesthesia with isoflurane, which could induce an inflammatory response^22,23^.

In agreement with our previous studies, the combined fracture and thoracic trauma resulted in a pulmonary inflammation in WT mice, as confirmed by increased C3a, IL-6 and chemokine (C-X-C motif) ligand 1 (CXCL1) concentrations (Fig. 2b–d)^21,24^. In the absence of C5aR1, only IL-6 was significantly increased, whereas other mediators were unaffected, indicating reduced pulmonary inflammation. In contrast, in C5aR2^−/−^ mice, the combined trauma increased almost all inflammatory mediators in the BAL fluids compared to C5aR2^−/−^ mice that received only an isolated fracture. Moreover, the levels of IL-1β, TNFα, IL-10, CXCL1 and MCP-1 were significantly elevated compared to WT mice, indicating a more pronounced inflammatory response (Fig. 2b–d).

#### Fracture haematoma/callus

**Figure 3:**
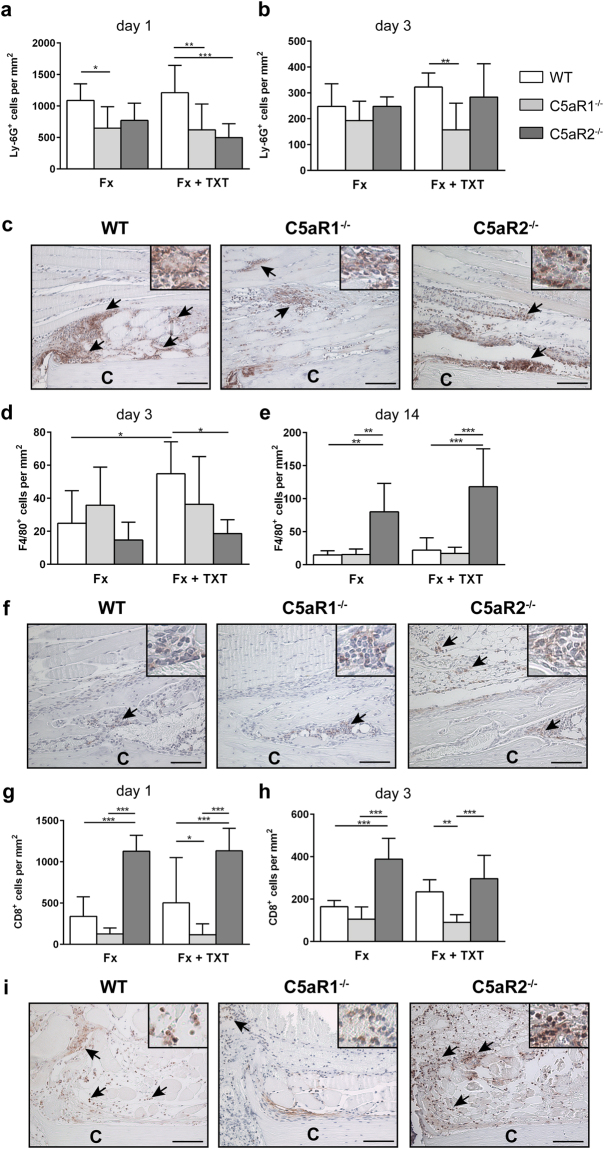
Quantification and representative immunohistological staining of immune cells in the fracture haematoma/callus. Quantitative analysis of Ly-6G^+^ neutrophils in the fracture haematoma 1 (**a**) and 3 (**b**) days after trauma calculated in the periosteal area between the two inner pins of the external fixator. Representative neutrophil staining at day 1 (**c**). Quantitative analysis of F4/80^+^ macrophages in the fracture haematoma and callus calculated either in the endosteal area in direct proximity to the fracture gap 3 days after trauma (**d**) or in the periosteal callus 14 days after trauma (**e**). Representative macrophage staining at day 14 (**f**). Quantitative analysis of CD8^+^ cells in the fracture haematoma 1 (**g**) and 3 (**h**) days after trauma calculated in the periosteal area between the two inner pins of the external fixator. Representative staining of T-cells at day 1 (**i**). Fx: mice with isolated fracture, Fx + TXT: mice with combined fracture and thoracic trauma. n = 6, scale bar 100 µm. *p < 0.05, **p < 0.01, ***p < 0.001.

In the absence of C5aR1, the recruitment of neutrophils and T-cells was significantly reduced 1 and 3 days after injury (Fig. 3a–c and g–i). These effects were more prominent in the combined trauma group than in the group with an isolated fracture. Macrophage recruitment was unaffected in C5aR1^−/−^ mice (Fig. 3d–f) either in the isolated fracture group or after an additional thoracic trauma.

In C5aR2^−/−^ mice, neutrophil recruitment was impaired only in the combined trauma group 1 day after injury. Notably, the recruitment of T-cells in these mice was significantly increased both 1 and 3 days after isolated fracture and after combined trauma (Fig. 2g–i). Macrophage recruitment in C5aR2^−/−^ mice was unaffected compared to WT mice 3 days after injury, but was significantly increased 14 days after fracture both in the isolated fracture group and after combined trauma, indicating a prolonged immune response (Fig. 3d–f).

### Fracture healing in C5aR1^−/−^ and C5aR2^−/−^ mice

**Figure 4:**
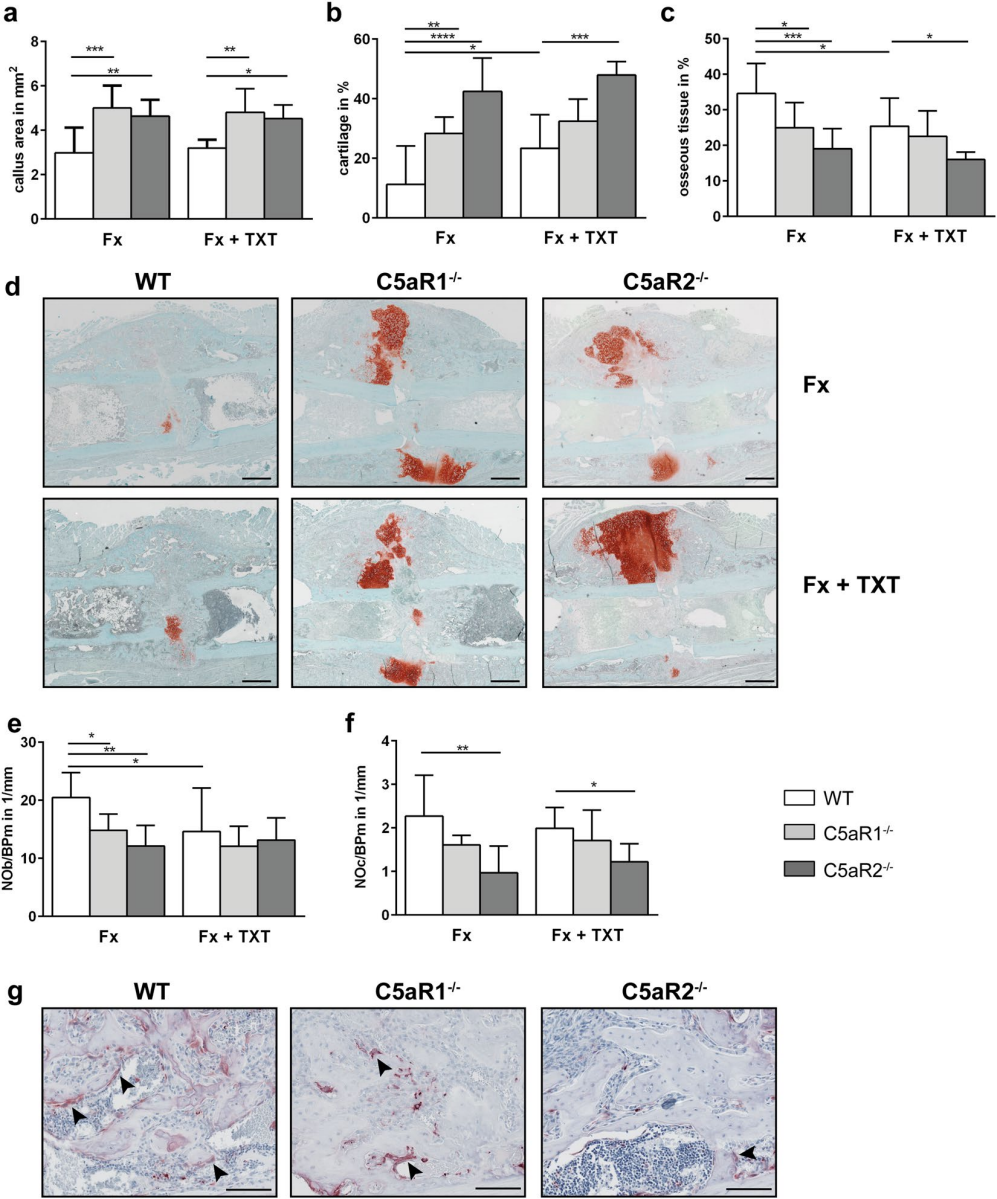
Analysis of fracture healing 14 days after trauma. Areas of different tissues as determined by Safranin O staining: total callus area (**a**), relative cartilage content (**b**) and osseous tissue content (**c**). Representative images of Safranin O-stained femurs at day 14 (**d**). Scale bar 500 µm. Relative numbers of osteoblasts (**e**) and osteoclasts (**f**) in the periosteal fracture callus. Representative images of TRAP staining at day 14 (**g**). Scale bar 100 µm. Fx: mice with isolated fracture, Fx + TXT: mice with combined fracture and thoracic trauma. n = 5–6, *p < 0.05, **p < 0.01, ***p < 0.001.

During endochondral ossification 14 days after isolated fracture, both C5aR1^−/−^ and C5aR2^−/−^ mice displayed a significantly larger callus with increased cartilage and decreased bone contents, indicating disturbed endochondral bone formation (Fig. 4a–d). This could be explained by delayed cartilage-to-bone transformation, because in both mouse strains the numbers of osteoblast in the fracture callus were significantly reduced. Osteoclast numbers were significantly diminished only in the absence of C5aR2 (Fig. 4e–g). In the combined trauma group, the absence of C5aR1 and C5aR2 provoked similar effects.

**Figure 5:**
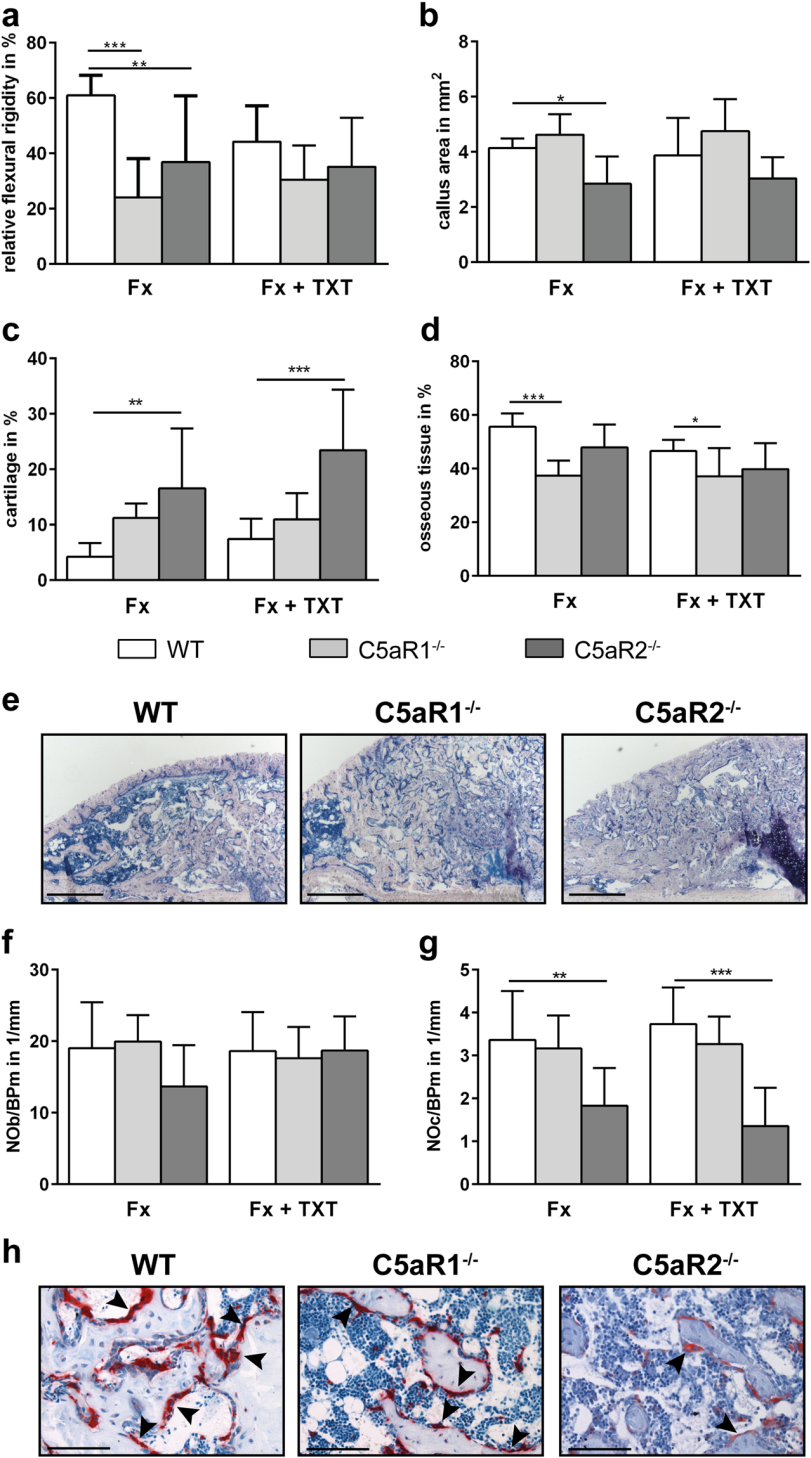
Analysis of the fracture healing 21 days after trauma. Relative bending stiffness of the fracture callus (**a**). Areas of different tissues as determined by Giemsa staining: total callus area (**b**), relative cartilage content (**c**) and osseous tissue content (**d**). Representative images of fracture callus stained with Giemsa (**e**). Scale bar 500 µm. Relative numbers of osteoblasts (**f**) and osteoclasts (**g**) in the periosteal fracture callus. Representative images of TRAP staining at day 21 (**h**). Scale bar 100 µm. Arrowheads indicate osteoclasts. Fx: mice with isolated fracture, Fx + TXT: mice with combined fracture and thoracic trauma. n = 6–8, *p < 0.05, **p < 0.01, ***p < 0.001.

Both C5aR1^−/−^ and C5aR2^−/−^ mice 21 days after isolated fracture displayed impaired fracture healing, as demonstrated by a significantly reduced bending stiffness of the fracture callus (Fig. 5a). In the absence of C5aR1, the bone content in the fracture callus was significantly decreased, whereas all other parameters were not significantly affected (Fig. 5b–d). C5aR2^−/−^ mice displayed a significantly smaller callus, persistent cartilage and an increased osteoclast number (Fig. 5b,c,g,h). In agreement with our previous studies^21,24^, the additional thoracic trauma impaired fracture healing in WT mice, as indicated by a decreased flexural rigidity (p = 0.07) and bone content (p = 0.058). In C5aR1^−/−^ and C5aR2^−/−^ mice, the additional thoracic trauma did not further affect fracture healing.

## Discussion

The present study addressed the role of C5aR1 and C5aR2 in fracture healing using respective knockout mouse models. The early inflammatory response and bone repair were investigated in an isolated fracture model and in a combined model of fracture and thoracic trauma associated with a more pronounced inflammation. C5aR1^−/−^ mice displayed reduced systemic and pulmonary inflammation, a reduced recruitment of neutrophils to the fracture site, disturbed bone formation and persistent cartilage. In the absence of C5aR2, the immune response in the lungs and fracture haematoma was more pronounced and endochondral ossification was considerably impaired. These results suggest that the receptors differentially regulate the immune response after fracture, whereas during later healing stages both C5aR1 and C5aR2 stimulate osteoclast formation and cartilage-to-bone transformation.

First, we analysed the bone phenotype of C5aR1^−/−^ and C5aR2^−/−^ mice. It is known that complement proteins, and particularly C5aR1, are expressed by various bone-cell populations, including mesenchymal stem cells, chondroblasts, osteoblasts and osteoclasts^9,10^. To the best of our knowledge, C5aR2 has to date not been investigated in bone. C5aR1^−/−^ mice exhibited an increased cortical and trabecular bone mass. The bone formation rate and the serum levels of the bone formation marker PINP were significantly elevated, however osteoblast numbers were not significantly affected in C5aR1^−/−^ mice. Interestingly, these mice demonstrated significantly reduced osteoclast numbers *in vivo*. *Ex vivo* analysis of cells derived from these mice also demonstrated that osteoclastogenesis was considerably disturbed, indicating that the increased bone mass might be due to disturbed osteoclast function. In contrast, osteoblast proliferation and activity were unaffected in the absence of C5aR1. This is in agreement with other studies demonstrating that C5a induces osteoclast formation directly and indirectly by increasing receptor activator of NF-κB ligand (RANKL) expression, a key osteoclastogenesis stimulator^12,13^. C5aR2^−/−^ mice also displayed an increased bone mass, albeit the phenotype was less pronounced. Moreover, in contrast to C5aR1^−/−^ mice, osteoclast formation was unaffected both *in vivo* and *in vitro*. Therefore, the increased bone mass of C5aR2^−/−^ mice may rather result from the higher osteoblast numbers observed *in vivo*, although the activity of osteoblasts isolated from these mice was unaffected, suggesting that osteoblasts do not display a cell-autonomous defect. The slightly increased osteoblast numbers *in vivo* could be explained by an increased recruitment, because C5a regulates MSC and osteoblast migration^14,25^. Furthermore, we cannot exclude an indirect effect, because we used general knockout mice. In summary, our results confirm previous data regarding the crucial regulatory role of C5aR1 in osteoclastogenesis^12,13^ and, furthermore, indicate that C5aR2 may also influence bone homeostasis.

To analyse the role of C5aR1 and C5aR2 in bone healing, we used both an isolated fracture model, which allows uneventful fracture healing without any significant systemic inflammatory response^21,26^; and a model of combined fracture and thoracic trauma, which provokes a more pronounced systemic inflammation^21,24,27^. Confirming our previous data, isolated fracture in the WT mice did not affect IL-6 serum levels, whereas combined trauma led to a significant increase of IL-6 concentrations.

C5aR1^−/−^ mice exhibited a significantly lower increase of serum IL-6 after the combined trauma compared to WT mice, confirming that the pulmonary inflammatory response after the additional thoracic trauma was also lower. Locally in the fracture haematoma, neutrophil recruitment in the absence of C5aR1 was significantly decreased, whereas macrophages were not significantly affected after both isolated fracture and after combined trauma. T-cell recruitment was non-significantly decreased in the absence of C5aR1, but did become significant after the additional thoracic trauma. Overall, these data indicate a pro-inflammatory role of C5aR1. This is in agreement with other inflammatory models, where the blockade of C5a or the absence of C5aR1 were protective in regard of the excessive inflammatory response, including severe thoracic trauma^28^, sepsis^29^ and acute lung injury^30^. However, the absence of C5aR1 did not completely abolish the systemic inflammatory response after combined injury, indicating that other pro-inflammatory mechanisms, including IL-6-mediated signalling, may also play an important role.

Notably, in C5aR2^−/−^ mice, both the isolated fracture and the combined trauma model led to increased pulmonary inflammation compared to WT mice, indicated by significantly elevated levels of TNFα, IL-1β, C3a and MCP-1 in the BAL fluids. These data are in accordance with Gerard *et al*., who observed significantly increased levels of TNFα and IL-6 in C5aR2^−/−^ mice after immune complex-mediated lung injury^31^, suggesting that these mice are more susceptible to inflammation. Furthermore, in C5aR2^−/−^ mice, we observed significantly increased T-cell recruitment to the fracture haematoma during the early inflammatory phase after both isolated fracture and combined trauma. Macrophage recruitment was also enhanced even 14 days after fracture, suggesting a stronger and prolonged immune response. These data indicate anti-inflammatory activity of C5aR2 in the context of bone healing.

The discrepancy in cytokine concentrations and immune cell recruitment between C5aR1^−/−^ and C5aR2^−/−^ mice could be explained by differential expression and distinct modes of action of these receptors. Thus, C5aR2 is usually described as a scavenger receptor for C5a, limiting its availability for C5aR1 activation^5,8,31^. Another function of C5aR2 is the formation of an active complex with C5aR1 and β-arrestin, which blocks C5aR1-mediated MAPK activation^6^. Through this, C5aR2 exhibits anti-inflammatory activity by inhibiting C5aR1-mediated cell activation. On the other hand, several studies described a pro-inflammatory activity of C5aR2 associated with MAPK-mediated increased release of inflammatory mediators, including high mobility group box 1 (HMGB1)^7,32,33^.

Normally, both receptors are simultaneously expressed on immune cells^5^. However, it was previously shown that expression levels vary between immune cell populations, thereby possibly executing different actions. On neutrophils, both C5aR1 and C5aR2 are abundantly expressed^6,32^. Because neutrophil recruitment in our study was disturbed in both C5aR1^−/−^ and C5aR2^−/−^ mice, we conclude that in the present model, both receptors on neutrophils might exert similar effects facilitating neutrophil recruitment to the inflammation site.

In contrast, C5aR1 expression appears much lower on monocytes and lymphocytes^34^, whereas C5aR2 expression was reported on different populations of lymphocytes, for example, NK cells^35^, iTregs^36^, monocytes and macrophages^32^. Here, one can speculate that in our model, C5aR2 on T-cells and macrophages does indeed function as a scavenger receptor and its complete absence strongly enhanced C5aR1-mediated effects, even if C5aR1 was expressed on a low level.

During endochondral ossification and the bone formation phase 14 and 21 days after injury, WT mice displayed uneventful healing after isolated fracture and delayed healing after additional thoracic trauma, associated with increased cartilage and decreased bone content in the callus, and subsequently impaired biomechanical properties, thereby confirming our previous studies^18,21,24^.

In C5aR1^−/−^ mice, isolated fracture resulted in impaired healing compared to WT mice already 14 days after injury, as indicated by a larger callus with significantly increased cartilage and decreased bone contents. This could be partly explained by the reduced inflammation in these mice. It is generally accepted that a balanced immune response may be important for bone repair, whereas either the reduction or the enhancement of inflammation disturbs bone regeneration after fracture^37–39^. In C5aR1^−/−^ mice the number of neutrophils in particular was reduced. Indeed, it was demonstrated that these cells play a crucial role in inducing down-stream responses leading to fracture repair^24,40^. Neutrophils can act pro-regenerative by removing cell debris, stimulating the switch of macrophages to an anti-inflammatory M2 phenotype or by depositing an “emergency extracellular matrix” consisting of fibronectin^24,40^. Another explanation for impaired healing in C5aR1^−/−^ mice could be delayed cartilage-to-bone transformation, because we observed significantly decreased osteoblast numbers, and, although osteoclast numbers were not significantly affected, their surface was significantly decreased (as determined by histomorphometry; data not shown). Indeed, previous *in vitro* studies demonstrated that C5aR-mediated signalling is required for osteoclastogenesis^12,13^. However, in the present study, restored osteoclast numbers could be attributed to the compensatory effects of other pro-inflammatory cytokines, which may also regulate osteoclastogenesis during the repair phase, including IL-6^41^. We still observed 21 days after injury a reduced osseous tissue area in the fracture callus and significantly reduced flexural rigidity. Similar effects were observed after the combined trauma, although to a lesser extent, because here the fracture healing was already impaired due to the absence of C5aR1.

The absence of C5aR2 affected fracture healing similarly to the C5aR1 knockout, although here the effects were more pronounced: we observed persistent cartilage associated with significantly decreased osteoclast numbers 14 and 21 days after injury. Similar effects were observed after combined injury with additional thoracic trauma. Impaired fracture healing could at least in part be due to the increased immune response after fracture observed in these mice. We found, *inter alia*, significantly more CD8^+^ T cells in the callus of these mice. In humans, CD8^+^ memory/effector T cells were identified as an indicator for delayed fracture healing, while the depletion of CD8^+^ cells improved healing in mice^42^. Furthermore, C5aR2 might play an important role in osteoclast formation, only under inflammatory conditions, because we did not detect any differences in osteoclast numbers in intact bone. Currently, we can only speculate about the underlying mechanisms. One possible explanation could be that C5aR2 supports C5aR1 functions in osteoclastogenesis, but is activated mainly under inflammatory conditions. It is known that the activities of the C5aRs depend on the concentration and type of their ligands (*i. e*. C5a and C5a-desArg, a C5a-degradation product)^4^. Because very limited C5a is generated under physiological conditions, C5aR1 signaling might prevail. However, under inflammatory conditions, C5a and C5a-desArg, which binds predominantly to C5aR2^4^, are abundantly generated. This may lead to strong C5aR2 activation, as already demonstrated in other cells^6,7^, thus increasing osteoclast formation. Another explanation for the reduced osteoclast numbers in the callus of C5aR2^−/−^ mice could be that the macrophage numbers were increased on days 14 and 21. In this phase, osteoclast formation normally increases to enable cartilage-to-bone transformation and callus remodeling. Macrophages and osteoclasts share the same precursor-cell source^43^. Possibly, macrophage development prevails because of the increased/prolonged immune response in C5R2^−/−^ mice. However, further studies are necessary to prove these hypotheses.

A limitation of this study might be that we used mice with a general knockout of either C5aR as a first approach. The rationale behind the selection of the mouse models was that fracture healing involves many cell types, including immune, bone and endothelial cells. To distinguish between bone cell-specific mechanisms, conditional knockout mice will be used in further experiments. Another limitation might be that we cannot exclude unknown contributions of different organs (*e. g*. the lungs and liver) to posttraumatic inflammation in the combined trauma model. However, fracture healing was similarly influenced in the isolated compared to the combined trauma group by the lack of the receptors, indicating that secondary effects may play a subordinate role.

In summary, our data clearly indicate that both complement receptors, C5aR1 and C5aR2, play an important role in bone homeostasis, affecting the osteoblast/osteoclast balance. Moreover, in the inflammatory phase of fracture healing, C5aR1 and C5aR2 exert distinct activities, presumably depending on their expression levels on different immune cell populations. However, during endochondral ossification, both receptors exert similar roles, affecting cartilage-to-bone transformation and cartilage degradation by osteoclasts. Therefore, we can conclude that the action of both complement anaphylatoxin C5a receptors is required throughout the entire course of fracture healing for a successful outcome.

## Materials and Methods

### Cell Culture

Primary osteoblasts were isolated from diaphyses of long bone of of 8–12-week-old mice and expanded in culture medium (α-MEM, Biochrom AG, Berlin, Germany) supplemented with 10% heat-inactivated fetal calf serum (FCS, Gibco, Darmstadt, Germany), 100 U/mL penicillin/streptomycin (Gibco), 1% L-glutamine (PAN-Biotech, Aidenbach, Germany) and 0.5% Fungizone^TM^ (Amphotericin B, Gibco) at 37 °C under 5% CO_2_ atmosphere. Cell proliferation was assessed using the colorimetric BrdU Cell Proliferation Assay Kit (Cell Signalling Technology, Danvers, MA, USA) according to manufacturer’s recommendations. For osteogenic differentiation, osteoblasts were seeded in 24-well plates (CELLSTAR®, Greiner Bio-One) at a density of 0.5 × 10^4^ cells/cm^2^. Differentiation was induced in presence of 0.2 mM ascorbate-2-phosphate and 10 mM β-glycerophosphate (both Sigma-Aldrich, Steinheim, Germany) for 14 days and reverse transcription and real-time quantitative PCR were performed. Isolation of total RNA and qPCR were performed as previously described^44^ using the following primers: *C5ar1* forward primer 5′-GGC CAT CCT GCG GCT GAT GG-3′, reverse primer 5′-GCC TTG CGA CTC CAG GTC CG-3′; *C5ar2* forward primer 5′-CAC ACC ACC AGC GAG TAT TAT G-3′, reverse primer 5′-AGC ACA AGC AGG ACT ATC AGG-3′; *Alpl* forward primer 5′-GCT GAT CAT TCC CAC GTT TT-3′, reverse primer 5′- GAG CCA GAC CAA AGA TGG AG-3′; *Bglap* forward primer 5′-GCG CTC TGT CTC TCT GAC CT-3′, reverse primer 5′ ACC TTA TTG CCC TCC TGC TT-3′; *Ibsp* forward primer 5′-GAA GCA GGT GCA GAA GGA AC-3′, reverse primer 5′-GAA ACC CGT TCA GAA GGA CA-3′. Gene expression was analysed relative to the housekeeping gene glyceraldehyde 3-phosphate dehydrogenase (*Gapdh* forward primer 5′-ACC CAG AAG ACT GTG GAT GG -3′, reverse primer 5′-GGA TGC AGG GAT GAT GTT CT-3′) and then normalized to undifferentiated control. The capillary electrophoresis of PCR products was performed using the QIAxcel Advanced System (Qiagen, Hilden,Germany).

Osteoclast precursor cells were extracted from bone marrow and cultured for 3 days in culture medium (α-MEM, Biochrom) with 10% FBS Superior (Biochrom), 100 U/mL penicillin/streptomycin (Gibco), 1% L-glutamine (PAN-Biotech) and 0.5% Fungizone^TM^ (Gibco) and supplemented with 35 ng/mL macrophage colony-stimulating factor (M-CSF, EMD Millipore Corporation, Darmstadt, Germany). Non-adherent cells (5 × 10^5^/cm^2^) were further cultured for 13 days in 96-well plates in culture medium supplemented with 25 ng/mL recombinant human M-CSF (EMD Millipore Corporation) and 50 ng/mL recombinant mouse RANKL (R&D Systems, Wiesbaden, Germany). TRAP staining (TRAP staining kit, Sigma) and terminal deoxynucleotidyl transferase dUTP nick end labeling (TUNEL assay, CF488A TUNEL assay apoptosis detection kit, Biotium) were performed according to the manufacturer’s protocol. TRAP-positive cells with more than 2 nuclei were counted as osteoclasts.

### Animal experiments

All animal experiments were performed according to the international regulations for the care and use of laboratory animals and were approved by the local Ethical Committee (No. 1096, Regierungspräsidium Tübingen, Germany). Male C5aR1- (C5aR1^−/−^) and C5aR2-knockout (C5aR2^−/−^) mouse models were originally established by the group of C. Gerard^31,45^. Corresponding control WT C57BL/6 J mice were purchased from Charles River (Sulzfeld, Germany). All mice received a standard mouse feed (ssniff^®^ R/M-H, V1535-300, Ssniff, Soest, Germany) and water *ad libitum*.

To investigate whether C5aR1 or C5aR2 knockout influences bone formation, the skeletons of male 12-week-old WT and knockout mice were analysed by µCT and histomorphometry as described below. All mice received subcutaneous injections of alizarin red and calcein green (30 mg/kg body weight) (both from Sigma, Steinheim, Germany) 3 and 12 days, respectively, before bone harvesting to determine the bone formation rate. Additionally, bone turnover markers were determined in serum using RatLaps^TM^ (CTX-I) EIA and Rat/Mouse PINP EIA (both from Immunodiagnostic Systems GmbH (IDS GmbH), Frankfurt am Main, Germany) according to manufacturer’s protocols.

To assess fracture healing, 12-week-old mice received a femur osteotomy (0.4 mm) stabilised using an external fixator (RISystem, Davos, Switzerland) under general anaesthesia with 2% isoflurane (Forene, Abbott, Wiesbaden, Germany)^21,26^. Additionally, 50% of the mice received an additional thoracic trauma immediately after fracture while still under general anaesthesia. In brief, a single blast wave was applied to the middle of the thorax using a blast-wave generator. This model produces a standardized bilateral, isolated lung contusion^46^. For pain medication, all mice received 25 mg/L tramadol hydrochloride (Tramal®, Gruenenthal GmbH, Aachen, Germany) in the drinking water from 1 day pre-surgery until 3 days post-surgery. Animals were sacrificed at 3 h and 1, 3, 14 and 21 days after trauma using an isoflurane overdose and terminal cardiac puncture with blood withdrawal. A total of 5–8 mice were used per group at each time point.

### Tissue preparation

Blood was collected 3 h after trauma in microvettes (Microvette^®^ 100 µL, coated with coagulation activator, Sarstedt AG & Co, Nümbrecht, Germany) and centrifuged at 4000 × *g* for 10 min. The serum was collected and stored at −80 °C.

Osteotomized femurs from days 1, 3 and 14 after trauma were immediately fixed in 4% buffered formalin for immuno- and histochemical staining. For biomechanical testing on day 21, intact and fractured femurs were stored in 0.9% NaCl (B. Braun Melsungen AG, Melsungen, Germany) during measurement to avoid sample dehydration and then fixed in 4% buffered formalin for histological analysis. Additionally, for phenotyping, lumbar spine (L2–L5) was harvested and immediately fixed in 4% buffered formalin.

### Cytokine analysis

Serum IL-6 concentration was determined using a mouse ELISA kit (IL-6 BD Biosciences, Singapore, 1:150). Anaphylatoxin and cytokine concentrations in BAL fluid were determined using a mouse ELISA kit (C3a and C5a: R&D Systems) and a mouse multiplex cytokine kit (Bio-Plex Pro Cytokine Assay, Bio-Rad, Hercules, CA), respectively, according to the manufacturers’ protocols. Data were analysed using the standard curve of cytokine standards (Bio-Plex Manager Software 4.1).

### Histomorphometry and immunohistochemistry

For bone phenotyping, femurs and lumbar spine were fixed in 4% buffered formaldehyde and embedded in polymethylmethacrylate. OsteoMeasure image analysis software (OsteoMetrics Inc., Decatur, GA) was used to determine the number of osteoblasts in toluidine blue-stained sections, the number of osteoclasts in tartrate-resistant acid phosphatase (TRAP)-stained sections and the bone formation rate/bone surface (BFR/BS) according to the guidelines of the American Society for Bone and Mineral Research (ASBMR).

Osteotomized femurs were decalcified using 20% ethylenediaminetetraacetic acid (EDTA, pH 7.2–7.4) and embedded in paraffin. For histomorphometry, 6 µm-thick femur sections were stained using Safranin O or Giemsa. All femurs were analysed by light microscopy (Leica DMI6000B, Leica, Heerbrugg, Switzerland) under 50-fold magnification. The periosteal callus between the two inner pins was defined as a region of interest (ROI). The relative amounts of osseous tissue and cartilage were evaluated by image analysis (MMAF Version 1.4.0 MetaMorph^®^, Leica). The different immune-cell populations were stained using the following antibodies and dilutions: neutrophil granulocytes: 1:300 LEAF™ anti-mouse Ly-6G antibody (#127620, BioLegend, Munich, Germany) and 1:200 goat anti-rat immunoglobulin G (IgG) secondary antibody (#A10517, Life Technologies GmbH, Darmstadt, Germany); macrophages: 1:500 rat anti-mouse F4/80 antibody (#MCA497GA, AbD Serotec, Puchheim, Germany) and 1:200 goat anti-rat IgG secondary antibody (#A10517, Life Technologies GmbH, Darmstadt, Germany); T-cells and dendritic cells: 1:500 rabbit anti-CD8 polyclonal antibody (#bs-0648R, Bioss Inc, Woburn, MA) and 1:200 goat anti-rabbit IgG secondary antibody (#B2770, Life Technologies GmbH). Respective non-specific IgG antibodies were used as controls. Subsequently, the slides were incubated with avidin-biotin complex (Vector Laboratories Inc., Burlingame, CA) for signal amplification. NovaRed (Vector Laboratories Inc.) was used as a chromogen. All sections were counterstained using haematoxylin (Waldeck, Münster, Germany) and analysed under 200- or 400-fold magnification by light microscopy.

### Biomechanical testing of the femurs

To evaluate the mechanical competence of the healed femurs, the flexural rigidity of the femurs explanted on day 21 was investigated using a non-destructive three-point bending test on a material-testing machine (1454, Zwick GmbH, Ulm, Germany). Briefly, an axial load was applied to the femoral midshaft, with a maximum force of 4 N. The flexural rigidity (EI) was calculated from the load-deflection curve as described previously^21,26^.

### Micro-computed tomography (µCT)

For bone phenotyping, lumbar spine and femurs were scanned using a µCT device (Skyscan 1172, Bruker microCT, Kontich, Belgium) at 8-µm resolution using 50 kV and 200 mA^21^. µCT analysis was performed using Skyscan software (NRecon, DataViewer, CTAn, all from Bruker microCT). Calibration and thresholding (394 mg hydroxyapatite/cm^3^ for trabecular bone and 642 mg hydroxyapatite/cm^3^ for cortical bone) were performed in accordance with the ASBMR guidelines for µCT analysis as described previously^47,48^. For bone phenotyping, the volumes of interest (VOIs) were defined as following: in the cortical bone a VOI of 168 µm height was set in the femur mid-diaphysis; for the trabecular bone a cylindrical VOI was set between the two endplates of L4.

### Statistical analysis

The results are presented as the mean ± standard deviation. All data were analysed in a blinded fashion. Statistic software GraphPad Prism 6 (GraphPad Software, La Jolla, CA) was used to evaluate the data. Groups were tested for normal distribution using the Shapiro-Wilk test, then compared by one-way analysis of variance (ANOVA) and Fisher’s LSD *post-hoc* test. The level of significance was set to p < 0.05.
